# Real-world treatment patterns and outcomes among patients with HER2-positive unresectable or metastatic breast cancer in China

**DOI:** 10.3389/fonc.2025.1527990

**Published:** 2025-05-14

**Authors:** Jiajia Huang, Limin Chen, Lihua Song, Zhongsheng Tong, Tao Sun, Xiaojia Wang, Yi Liu, Shusen Wang

**Affiliations:** ^1^ Department of Medical Oncology, State Key Laboratory of Oncology in South China, Guangdong Provincial Clinical Research Center for Cancer, Sun Yat-sen University Cancer Center, Guangzhou, China; ^2^ Department of Breast Medical Oncology, Shandong Cancer Hospital and Institute, Shandong First Medical University and Shandong Academy of Medical Sciences, Jinan, China; ^3^ Department of Breast Oncology, Tianjin Medical University Cancer Institute & Hospital, Tianjin, China; ^4^ Department of Breast Medicine 1, Cancer Hospital of China Medical University, Liaoning Cancer Hospital and Institute, Shenyang, China; ^5^ Department of Breast Cancer Internal Medicine, Zhejiang Cancer Hospital, Hangzhou Institute of Medicine (HIM), Chinese Academy of Sciences, Hangzhou, China; ^6^ Medical Affairs Department, Daiichi Sankyo (China) Holdings Co., Ltd., Shanghai, China

**Keywords:** metastatic breast cancer, HER2 overexpression, treatment pattern, effectiveness, real-world study

## Abstract

**Background:**

Breast cancer is now the most commonly diagnosed cancer in the world and the leading cause of cancer mortality in women worldwide. In past few years, anti- human epidermal growth factor receptor-2 (HER2) therapy for metastatic breast cancer (mBC) has rapidly altered in China. This study aimed to describe treatment patterns and outcomes in patients with HER2-positive unresectable or metastatic breast cancer in the real-world setting.

**Methods:**

This multicenter, retrospective analysis evaluated the treatment patterns and the efficacy in newly diagnosed HER2+ mBC patients between Jan 1, 2020 and Aug 31, 2022. Electronic medical records from 5 cancer centers in China were used.

**Results:**

Among 865 patients, the most common first-line (1L) treatment regimen was dual anti-HER2 blockade monoclonal antibody-based therapy (Dual anti-HER2 mAB: 36.07%), followed by single anti-HER2 blockade mAB-based therapy (Single anti-HER2 mAB: 21.97%) and single Tyrosine Kinase Inhibitor-based therapy (Single TKI: 19.19%). In the second-line (2L), the primary treatment was single TKI regimen (35.45%), followed by TKI+anti-HER2 blockade mAB-based therapy (TKI+anti-HER2 mAB: 16.36%) and single anti-HER2 mAB (15.15%). *De novo* mBC at initial diagnosis, recurrence post 6 months of (neo)adjuvant treatment, absence of brain metastasis, and younger age, were associated with the choice of dual anti-HER2 mAB regimen in 1L treatment. Conversely, patients receiving anti-HER2 therapy in (neo)adjuvant setting, having brain metastasis, and experiencing a recurrence within 6 months were more likely to receive TKI-based regimen. The median rwPFS of 1L and 2L treatment declined sequentially, with values of 11.04 [95% confidence interval (CI) 10.19–12.03] months and 7.59 (95% CI 6.21–9.20) months, respectively. Longer disease-free interval (DFI) and the choice of dual-anti HER2 regimen in 1L treatment were associated with longer rwPFS.

**Conclusion:**

The results of this study provide valuable real-world insight into HER2 positive mBC treatment trends and clinical outcomes, informing subsequent patient management.

## 1 Introduction

Breast cancer is a leading global health concerns with 2.26 million new cases reported in 2020. Notably, 416,371 of the newly diagnosed cases occurred in China and constituted 18% of the worldwide total number (1, 2). Among these cases, 6% to 30% were initially diagnosed with stage IV breast cancer or *de novo* metastatic breast cancer (mBC) (3), and nearly 30% of women diagnosed with early-stage breast cancer experienced metastasis (4). mBC was considered incurable, with a five-year survival rate of around 30% to 49% (5, 6).

Approximately 25% of breast cancer patients have an amplification of the human epidermal growth factor receptor-2 (HER2) expression in China (7, 8), which is associated with aggressive tumor behavior (9). Anti-HER2 therapy, including anti-HER2 antibodies (trastuzumab, pertuzumab, margetuximab), tyrosine kinase inhibitors (TKIs) (lapatinib, pyrotinib, neratinib, tucatinib), and antibody-drug conjugates (ADCs) (trastuzumab-emtansine [T-DM1], trastuzumab deruxtecan[T-DXd]), have improved patient outcomes (10, 11). Currently, the global standard of care for HER2-positive (HER2+) mBC includes pertuzumab plus trastuzumab and chemotherapy for first-line (1L) treatment and T-DXd for second-line (2L) (10, 12–14). The optimal choice of third-line (3L) treatment options, such as T-DM1, the combination of tucatinib plus trastuzumab and capecitabine, neratinib plus capecitabine, and magertuximab plus chemotherapy, continues to be a topic of debate.

However, the treatment landscape in China differs (15). Pyrotinib, an oral TKI, plays a crucial role in managing HER2+ mBC in China. The PHOEBE trial demonstrated the superior efficacy of pyrotinib plus capecitabine over lapatinib plus capecitabine, establishing it as the preferred choice after trastuzumab (16). The recently released PHILIA trial demonstrated longer median progression-free survival (mPFS) with pyrotinib, trastuzumab and taxane than trastuzumab alone with taxane, leading to the combination of pyrotinib, trastuzumab and taxane being considered in HER2+ mBC 1L treatment (17). Moreover, the rapid approval of multiple anti-HER2 agents in mBC further contributes to the evolving landscape of anti-HER2 therapy in Chinese breast cancer treatment.

The diverse array of treatment options in China has resulted in a growing divergence in clinical practices for breast cancer management compared to global standards. However, the real-world data on their use and efficacy in China is limited, especially in newly established treatment sequences following the approval of novel drugs. Meanwhile, the 1L standard of care (SOC) was based on the CLEOPATRA study conducted a decade ago, but had encountered challenges from real-world clinical practice, including the widespread adoption of pertuzumab in adjuvant therapy and the absence of a head-to-head comparison between pyrotinib and pertuzumab regimens in 1L. Therefore, there is a crucial need for real-world data to fill this gap by examining the treatment pattern and effectiveness of HER2+ mBC therapies in China, providing data for further individualized management of patients with HER2+ mBC.

## 2 Methods

### 2.1 Study design and patients

This study was a multicenter, non-interventional, retrospective study conducted in five cancer centers across China. The study included patients aged over 18 who were newly diagnosed with HER2-positive breast cancer that was unresectable or metastatic between January 1, 2020 and August 31, 2022, and who had received at least one round of systemic treatment. Patients with other malignancies, or participation in unblinded clinical trials were excluded. The de-identified patient data were retrospectively derived from electronic medical records (EMRs). This study has been registered on ClinicalTrials gov (NCT05769751).

### 2.2 Outcomes measures

The primary outcome was real-world treatment patterns, defined as the distribution and sequence of various systemic therapy regimens across different treatment lines. The secondary outcome was, real-world progression-free survival (rwPFS), assessed the time from the commencement of the current treatment line to documented disease progression or death, whichever occurred first. rwPFS data for patients without disease progression or death as of the last follow-up date were censored at the time of the last tumor imagining evaluation. A line of treatment was defined as one regimen, possibly a combination of several drugs, given from the date of initiation until failure to control the disease, treatment discontinuation, changing of anti-HER2 drugs, or patient participation in a clinical trial. Other study variables collected included demographic and clinical-pathological characteristics (age, gender, metastatic lesions, Eastern Cooperative Oncology Group Performance status, hormone receptor status, HER2 status, prior therapies), as well as post-treatment outcomes (disease progression, subsequent treatments, vital status and date of last known contact/death).

### 2.3 Statistical analysis

Descriptive analyses were performed on demographic and clinical characteristics, treatment patterns, and treatment outcomes in the overall population and in subgroup of patients with brain metastasis. Number and percentage of patients were calculated for dichotomous and polychotomous variables. Means, standard deviations (SDs), medians, interquartile ranges (IQRs), minimum and maximum were provided for continuous variables. The distribution of 1L treatment choices in patient groups categorized by their time-to-relapse after completing anti-HER2 neo(adjuvant) therapy were compared using Fisher’s exact test. Sankey diagrams were created to visualize the sequence of systemic therapy regimens between treatment lines. Logistic regression analysis was performed to identify the factors influencing first-line therapy choices, with the quantitative association represented by odds ratios (ORs) with 95% confidence intervals (CIs). rwPFS along was estimated using the Kaplan-Meier methods and the survival curve was plotted. Cox proportional hazard models were used to evaluate factors impacting treatment outcomes. Variables included in the final multivariate model were selected according to the statistical significance in univariate analysis (cut-off *p valu*e < 0.05). Results are presented as hazard ratios (HRs) with 95% CIs. Statistical significance was set at a two-tailed *p value* < 0.05. All analyses were conducted using R software (Version 4.2.1s).

## 3 Results

### 3.1 Patients characteristics

A total of 865 patients with newly diagnosed unresectable or metastatic breast cancer were included in the study. The median age of the patients was 53 years (IQR, 45.67–58.54 years), and 12.02% (n = 104) were aged ≥65 years. More than half of the patients were postmenopausal (55.72%). 62.89% of the patients were diagnosed with recurrent mBC, while 37.11% were *de novo* mBC. Of the 861 patients with metastases, 63.65% had visceral metastases, and 54.70% had more than 1 metastatic site. The most common metastatic sites were bone (n = 366, 42.31%) and lung (n = 344, 39.77%), followed by liver (n = 304, 35.14%) and brain (n = 95, 10.98%). At baseline, the HR status was positive in 322 (54.03%) patients, with HER2 3+ accounting for 74.33% (n = 443) of them. The median follow-up time was 12.84 months (IQR: 6.08–19.96 months). A complete summary of patient characteristics was presented in **Table 1**.

**Table 1:** Patient and disease characteristics.

Characteristics	Value (N = 865)
Age (years)	
Mean (SD)	52.37 (10.52)
Median [Q1, Q3]	53.00 [45.67, 58.54]
Min, Max	21.87, 86.30
Age group (years), No. (%)	
<35	63 (7.28)
[35,65)	698 (80.69)
≥65	104 (12.02)
Sex, No. (%)	
Female	865 (100.00)
Family history of breast cancer, No. (%)	
Yes	76 (8.79)
No	657 (75.95)
Unknown	132 (15.26)
Menopausal status, No. (%)	
Post-menopausal	482 (55.72)
Pre-menopausal	314 (36.30)
Unknown	69 (7.98)
Eastern Cooperative Oncology Group performance status, No. (%)	
0-1	495 (57.23)
2-3	17 (1.97)
Missing	353 (40.81)
Disease history at initial diagnosis, No. (%)	
*De novo* mBC	321 (37.11)
Recurrent mBC	544 (62.89)
Time from the end of (neo)adjuvant treatment to recurrence by group, No. (%)	
<6 months	120 (26.79)
≥6 months	328 (73.21)
Site of primary lesion, No. (%)	
Left breast	447 (51.68)
Right breast	402 (46.47)
Bilateral breasts	16 (1.85)
Distant metastasis, No. (%)	
Yes	850 (98.20)
No	15 (1.80)
Visceral Disease, No. (%)	
Yes	548 (63.65)
No	313 (36.35)
Number of metastatic sites, No. (%)	
1	390 (45.30)
2	251 (29.15)
≥3	220 (25.55)
Selected metastatic sites, No. (%)	
Bone	366 (42.31)
Lung	344 (39.77)
Liver	304 (35.14)
Brain	95 (10.98)
HR status^a^, No. (%)	
Positive	458 (53.26)
Negative	388 (45.12)
Undetected	14 (1.63)
HER2 status^a^, No. (%)	
0	6 (0.70)
1+	6 (0.70)
2+	208 (24.19)
3+	622 (72.33)
Missing	18 (2.09)
Follow-up (months)	
Mean (SD)	13.56 (8.54)
Median [Q1, Q3]	12.84 [6.08, 19.96]
Min, Max	0.05, 32.02

a The most closely pathological result from the baseline. mBC, metastatic breast cancer; HR, hormone receptor; HER2, human epidermal growth factor receptor 2.

### 3.2 Treatment patterns and associated influential factors

**Figure 1:**
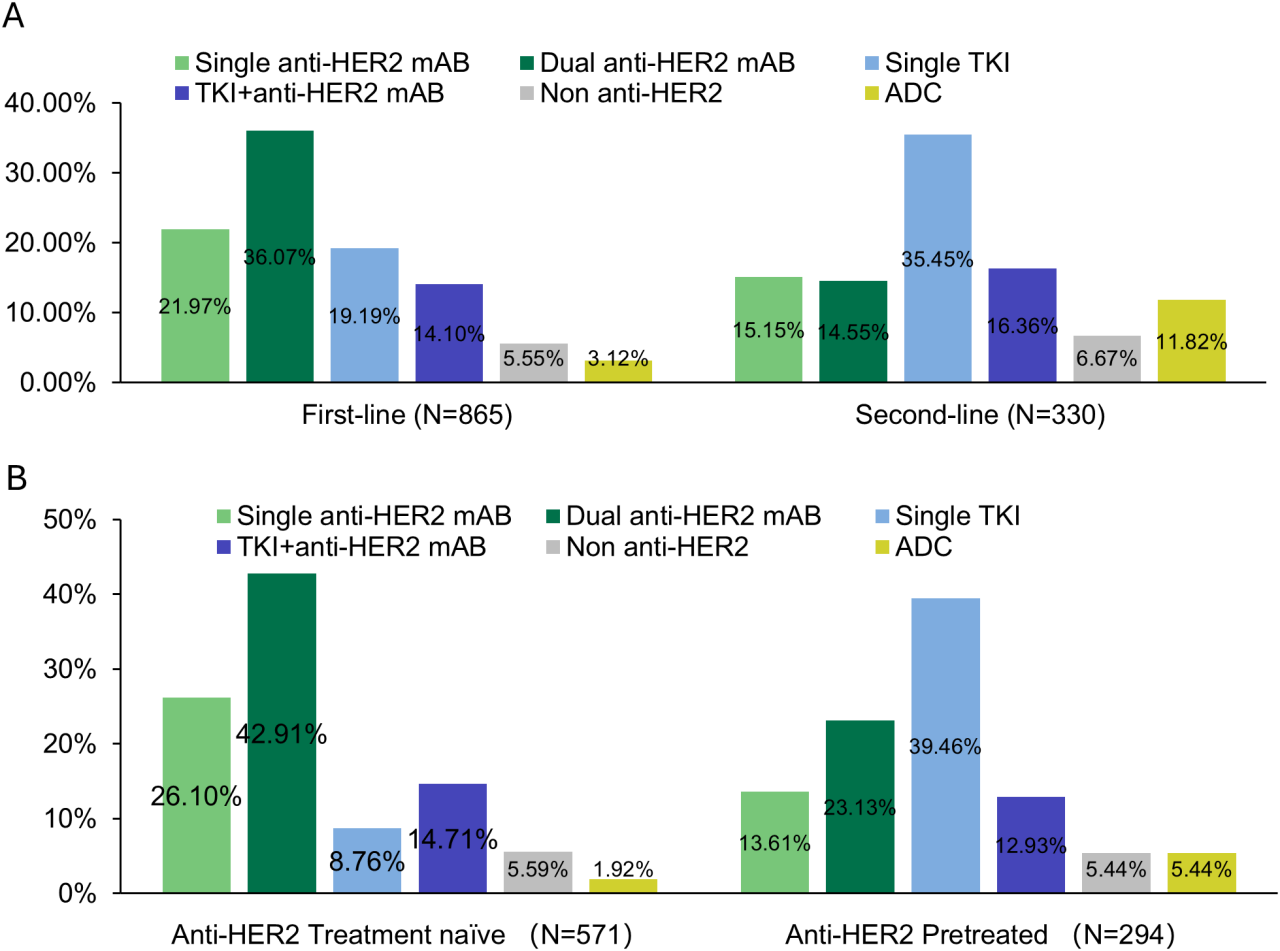
Treatment patterns: **(A)** across first-line and second-line, and **(B)** treatment patterns in first-line among anti-HER2 treated and naïve patients.

**Figure 2:**
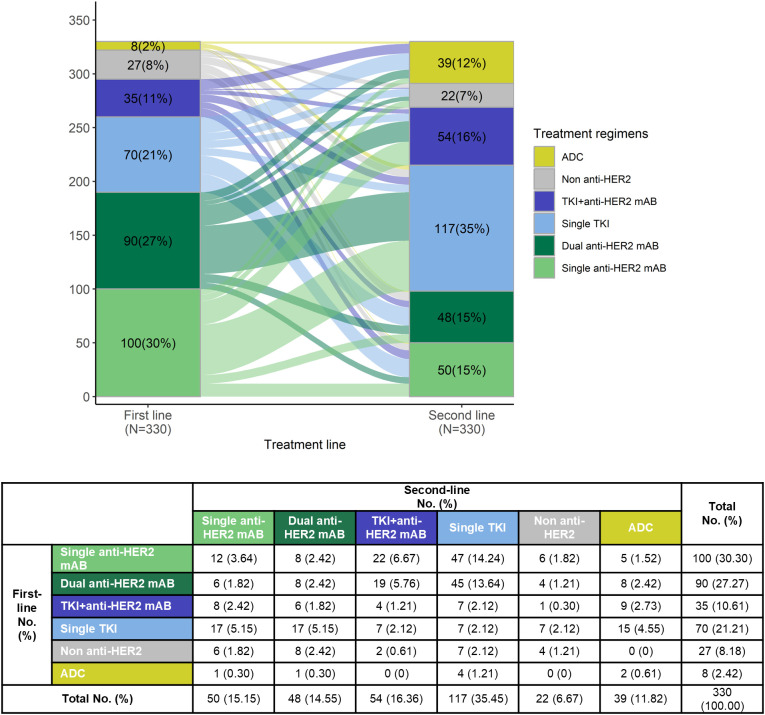
Treatment sequence from first-line to second-line treatment among patients who received at least two lines of systemic treatment.

Regarding the proportion of each systemic therapy regimen across treatment lines, 865 (100.00%) received at least 1 line of systemic therapy for mBC. In our data set, 38.15% had 2L treatment information, and 15.03% had post-2L treatments. Considering the complexity of real-world usage, the treatment regimens were categorized into six groups based on the type of anti-HER2 therapy: dual anti-HER2 blockade monoclonal antibodies (Dual anti-HER2 mAB), single anti-HER2 blockade mAB-based therapy (Single anti-HER2 mAB), single Tyrosine Kinase Inhibitor (TKI)-based therapy (Single TKI), TKI + anti-HER2 blockade mAB-based therapy (TKI + anti-HER2 mAB), antibody-drug conjugates (ADCs), and non-anti-HER2 therapy. The prominent medications were trastuzumab (83.39% of single anti-HER2 mAB), trastuzumab plus pertuzumab (98.45% of dual anti-HER2 mAB), and pyrotinib (95.33% of single TKI). Detailed medications of each regimen can be found in **Supplementary Table S1**. 94.9% of the anti-HER2 mAB or TKI regimens utilized in the metastatic setting, and 96.7% in the 1L setting, were combined with chemotherapy (data not shown). The most common 1L treatment regimens were dual anti-HER2 mAB (36.07%), followed by single anti-HER2 mAB (21.97%), and single TKI (19.19%). In the 2L setting, single TKI was most common (35.45%), followed by TKI + anti-HER2 mAB (16.36%) and single anti-HER2 mAB (15.15%) (**Figure 1A**). Treatment selection in 3L and later lines showed considerable variability, as shown in **Supplementary Table S2**. Approximately 12.00% of the total patients included were enrolled in unblinded clinical trials in the real world (**Supplementary Table S3**). The transition patterns of anti-HER2 therapy from 1L to 2L were shown in **Figure 2**. The predominant treatment option following single or dual anti-HER2 mAB was TKI. Post-TKI therapy typically involved either anti-HER2 mABs (dual or single) or ADCs. The detailed regimen transition, as well as the corresponding medications, from 1L to 2L, and subsequently to 3L, were shown in **Supplementary Figure S1**.

### 3.3 Associated influential factors for 1L treatment choice

To understand the factors influencing physicians’ treatment decisions, univariate and multivariate analyses were conducted, focusing on the relationship between baseline characteristics and 1L treatment regimens selection. Two comparisons based on predominant 1L and 2L treatment options were included in the analysis: TKI-based vs. non-TKI therapies and dual mAB vs. non-dual mAB therapies. Variables with statistically significance association on univariate analysis (**Supplementary Table S4**) were included in the multivariate logistic regression model (**Table 2**). Results of the multivariate analysis showed that the likelihood of opting for TKI-based regimen over non-TKI therapies in 1L was significantly higher in patients previously treated with anti-HER2 therapy in (neo)adjuvant setting [vs. HER2 treatment naïve, OR: 2.43 (1.61–3.69), *p* < 0.001], those with brain metastasis [vs. without brain metastasis, OR: 2.79 (1.76–4.46), *p* < 0.001], and patients who experienced a recurrence within 6 months [vs. ≥6 months, OR: 0.41 (0.26–0.65), *p* < 0.001] of post-(neo)adjuvant treatment. A higher preference for dual mAB regimen over non-dual mAB therapies in 1L was observed among patients with *de novo* mBC [recurrent mBC vs. *de novo* BC, OR: 0.32 (0.19–0.51), *p* < 0.001] at initial diagnosis, patients who experienced a recurrence post 6 months [vs. <6 months, OR: 2.60 (1.44–4.94), *p* = 0.002] of (neo)adjuvant treatment, those without brain metastasis [with vs. without brain metastasis, OR: 0.35 (0.18–0.61), *p* < 0.001], and patients aged less than 35 years [age ≥65 years vs. <35 years, OR: 0.48 (0.24–0.95), *p* = 0.04].

**Table 2:** Multivariate analysis for clinical factors of first-line treatment regimen choice.

Variables	TKI	Non-TKI	OR (95% CI)	*P* value	Variables	Dual mAB	Non-dual mAB	OR (95% CI)	*P* value
Disease history at initial diagnosis					Disease history at initial diagnosis				
*De novo* mBC	67	254	1.00	0.3	*De novo* mBC	184	137	1.00	<0.001
Recurrent mBC	221	323	1.34 (0.79, 2.24)		Recurrent mBC	128	416	0.32 (0.19, 0.51)	
Time from the end of (neo)adjuvant treatment to recurrence					Time from the end of (neo)adjuvant treatment to recurrence				
<6 months	77	43	1.00	<0.001	<6 months	16	104	1.00	0.002
≥6 months	116	212	0.41 (0.26, 0.65)		≥6 months	83	245	2.60 (1.44, 4.94)	
Previous treatment history					Prior anti-HER2 treatment				
Anti-HER2 treatment naïve	135	438	1.00	<0.001	Anti-HER2 treatment naïve	245	328	1.00	0.3
Anti-HER2 pretreated	153	139	2.43 (1.61, 3.69)		Anti-HER2 pretreated	67	225	1.26 (0.81, 2.00)	
Visceral disease					Visceral disease				
Yes	169	379	0.97 (0.71, 1.34)	0.9	Yes	199	349	0.74 (0.54, 1.02)	0.07
No	119	194	1.00		No	109	204	1.00	
Brain metastasis					Brain metastasis				
Yes	55	40	2.79 (1.76, 4.46)	<0.001	Yes	15	80	0.35 (0.18, 0.61)	<0.001
No	233	537	1.00		No	297	473	1.00	
					Age group, years				
					<35	33	30	1.00	NA
					[35,65)	241	457	0.58 (0.33, 1.03)	0.07
					≥65	38	66	0.48 (0.24, 0.95)	0.04

mBC, metastatic breast cancer; OR, odds ratio; CI, confidence interval; HER2, human epidermal growth factor receptor 2; NA, not applicable.

### 3.4 1L treatment choices by treatment history

Previous treatment history was a key factor in the choice of 1L therapy by physicians. **Figure 1B** showed the distribution of 1L treatment choices. Among the 571 patients naïve to anti-HER2 treatment, dual anti-HER2 mAB was the most common choice (42.91%, n = 245). As a comparison, of the 294 patients previously treated with anti-HER2 therapy, most (n = 116, 39.46%) opted for single TKI, followed by dual anti-HER2 mAB (n = 68, 23.13%). **Table 3** showed the 1L treatment choices in patients who were previously treated with anti-HER2 agents and relapsed. Of 217 patients who underwent single anti-HER2 mAB regimen in the (neo)adjuvant phase, 60.42% (n = 29 of 48) chose single TKI as 1L treatment following a relapse within 6 months. In contrast, for relapse occurring after more than 12 months, there was a higher preference for dual anti-HER2 therapy (37.88%, n = 50), followed by single TKI (23.48%, n = 31). In case of relapsing after dual anti-HER2 mAB, a higher preference for TKI was noted in 56.00% (n = 42 of 75) of the cases, regardless of prior efficacy. There was a significant difference in the distribution of 1L treatment choices in patients who received single anti-HER2 mAB (neo)adjuvant therapy (Fisher’s exact test, *p* < 0.001), but not in the group with dual anti-HER2 mAB (neo)adjuvant therapy (Fisher’s exact test, *p* = 0.09) (**Table 3**).

**Table 3:** First-line treatment regimens of patients who relapsed at different times after the completion of single/dual anti-HER2 mAB (neo)adjuvant regimen.

Single anti-HER2 mAB therapy group							Dual anti-HER2 mAB therapy group						
Time of relapse	No. (%)						Time of relapse	No. (%)					
	Total	<6 month	6-12month	≥12 month	Missing	*P* value		Total	<6 month	6–12 month	≥12 month	Missing	*P* value
	(N = 217)	(N = 48)	(N = 27)	(N = 132)	(N = 10)			(N = 75)	(N = 46)	(N = 10)	(N = 17)	(N = 2)	
First-line treatment choice							First-line treatment choice						
Single TKI	75 (34.56)	29 (60.42)	14 (51.85)	31 (23.48)	1 (10.00)	<0.001	Single TKI	42 (56.00)	30 (65.22)	5 (50.00)	7 (41.18)	0 (0)	0.09
Dual anti-HER2 mAB	61 (28.11)	4 (8.33)	1 (3.70)	50 (37.88)	6 (60.00)		ADC	9 (12.00)	4 (8.70)	2 (20.00)	3 (17.65)	0 (0)	
Single anti-HER2 mAB	33 (15.21)	3 (6.25)	4 (14.81)	25 (18.94)	1 (10.00)		TKI+anti-HER2 mAB	8 (10.67)	3 (6.52)	2 (20.00)	3 (17.65)	0 (0)	
TKI+anti-HER2 mAB	30 (13.82)	5 (10.42)	2 (7.41)	22 (16.67)	1 (10.00)		Dual anti-HER2 mAB	6 (8.00)	4 (8.70)	0 (0)	1 (5.88)	1 (50.00)	
Non anti-HER2	12 (5.53)	5 (10.42)	4 (14.81)	3 (2.27)	0 (0)		Single anti-HER2 mAB	6 (8.00)	2 (4.35)	0 (0)	3 (17.65)	1 (50.00)	
ADC	6 (2.76)	2 (4.17)	2 (7.41)	1 (0.76)	1 (10.00)		Non anti-HER2	4 (5.33)	3 (6.52)	1 (10.00)	0 (0)	0 (0)	

### 3.5 Real world efficacy of 1L and later line

**Figure 3:**
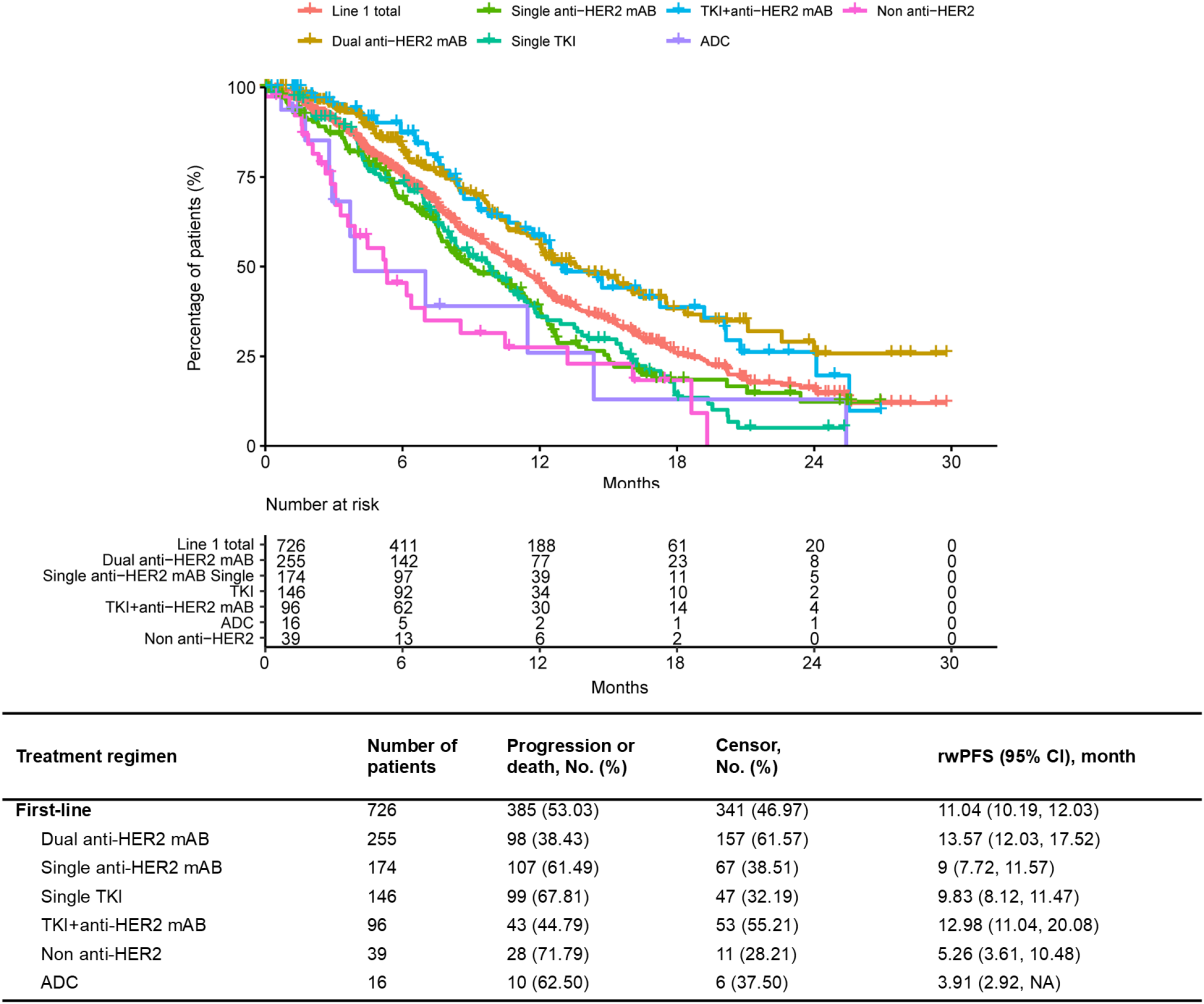
Progression-free survival of first-line treatment according to treatment regimens.

Of the 865 patients who received 1L treatment, the median rwPFS was 11.04 (95% CI 10.19–12.03) months. In terms of specific therapy regimen, the median rwPFS of patients treated with dual anti-HER2 mAB was 13.57 (95% CI 12.03–17.52) months, followed by TKI+anti-HER2 mAB with a median rwPFS of 12.98 (95% CI 11.04–20.08) months (**Figure 3**). The median rwPFS of each regimen in each treatment line after the 1L treatment were shown in **Supplementary Figure S2** and **Supplementary Table S5**. The longest estimated median rwPFS2 was observed among those who received a dual anti-HER2 mAB regimen in 1L and switched to a single TKI in 2L (24.32 months), albeit in a small sample (**Supplementary Table S6**).

### 3.6 Associated factors on real world outcome

The association between baseline characteristics, therapy regimens and treatment outcomes of 1L was further evaluated. We first performed a univariate analysis and identified several factors significantly associated with treatment outcomes, including dual anti-HER2 mAB vs. non-dual anti-HER2 mAB, prior exposure to dual anti-HER2 therapy, and time from the end of (neo)adjuvant treatment to recurrence (**Supplementary Table S7**). These factors were included in the multivariate cox regression model (**Table 4**), with treatments categorized as TKI vs. non-TKI and dual mAB vs. non-dual mAB. A longer median rwPFS was associated with patients who experienced a recurrence more than 6 months post-(neo)adjuvant treatment compared to those recurrence within 6 months, in both two analysis group (HR: 0.65, *p* = 0.008, adjusted for TKI-based regimen; HR: 0.70, *p* = 0.03, adjusted for dual mAB-based regimen). Additionally, patients who did not receive a dual anti-HER2 mAB regimen had a significantly higher risk of progression or death compared to those receiving a dual anti-HER2 mAB-based regimen (HR: 1.60, 95% CI: 1.26–2.05, P < 0.001).

**Table 4:** Multivariate analysis for clinical factors and the progression free survival of first-line treatment.

Variables	HR (95%CI)	*P* value	Variables	HR (95%CI)	*P* value
TKI-based regimen			Dual mAB-based regimen		
Non-TKI	1	0.6	Dual anti-HER2 mAB	1	<0.001
TKI	0.94 (0.75, 1.17)		Non dual anti-HER2 mAB	1.60 (1.26, 2.05)	
Disease history at initial diagnosis			Disease history at initial diagnosis		
*De novo* mBC	1	0.6	*De novo* mBC	1	0.4
Recurrent mBC	0.91(0.62, 1.33)		Recurrent mBC	0.86 (0.59, 1.26)	
Time from the end of (neo)adjuvant treatment to recurrence			Time from the end of (neo)adjuvant treatment to recurrence		
<6 months	1	0.008	<6 months	1	0.03
≥6 months	0.65 (0.48, 0.89)		≥6 months	0.70 (0.51, 0.96)	
Previous treatment history			Prior anti-HER2 treatment		
Anti-HER2 Treatment naïve	1	0.6	Anti-HER2 Treatment naïve	1	0.5
Anti-HER2 Pretreated	0.93 (0.69, 1.23)		Anti-HER2 Pretreated	0.91 (0.69, 1.22)	

HR, hazard ratio; CI, confidence interval; NA, not available; HER2, human epidermal growth factor receptor 2.

## 4 Discussion

The introduction and approval of a growing array of anti-HER2 drugs, including anti-HER2 antibodies (trastuzumab, pertuzumab), TKIs (lapatinib, pyrotinib, neratinib), and ADCs (Trastuzumab-emtansine, Trastuzumab deruxtecan), in recent years have substantially transformed the treatment landscape for HER2-positive breast cancer, leading to improved outcomes. Notably, pyrotinib, a unique medication developed in China, provided additional treatment options. However, there is a lack of comprehensive analysis and understanding of the real-world treatment patterns specific to China, as well as the treatment efficacy. This retrospective study provides the first comprehensive understanding of pre-treatment characteristics, real-world treatment patterns and their determinants, clinical outcomes and associated factors among patients diagnosed with HER2+ mBC in China. The results from this study can help to deepen the understanding of the treatment of HER2+ mBC, provide optimized treatment strategies for clinical decision making, and improve the prognosis of patients.

This study showed that the predominate choice in the 1L setting for HER2+ mBC was dual anti-HER2 mAB regimen (36.07%), in line with the Chinese guideline recommendations (18). However, the usage was lower compared to the other countries (44.3% and 68.7% reported in two studies) (19, 20). Several factors influence the lower use of 1L pertuzumab regimen. Firstly, it may be attributed to the late approval of pertuzumab for mBC in China in December 2019, following the results of PUFFIN study (21), while this retrospective study collected data on mBC patients diagnosed by 2020. Secondly, the low rate of pertuzumab adoption is challenged by the uptake of TKIs. The pyrotinib + capecitabine regimen was approved in 2018, indicated for use with or without prior trastuzumab treatment according to the 1L/2L study (22). The PHILA study further demonstrated the efficacy of pyrotinib in combination of trastuzumab in the 1L setting (17). The oral administration of pyrotinib and capecitabine provided the convenience for patients, particularly during the COVID-19 pandemic. Thirdly, considerations of real clinical characteristics contributed to the decision-making process. Thus, we explored potential factors influencing the choice of 1L regimen. *De novo* mBC diagnosis, compared to recurrent cases, was associated with a preference for dual mAB based regimen. For recurrent patients, the resistance to prior anti-HER2 treatment and the efficacy were be taken into consideration, which was similar with the China cross-sectional survey (23). Data has shown that previous anti-HER2 treatment could impact the efficacy of pertuzumab (6, 24). In addition, the length of the disease-free interval (DFI) had influence on the following regimens. A DFI of less than 6 months for trastuzumab may not warrant re-treatment with trastuzumab + pertuzumab, considered to be trastuzumab resistance. A recent phase II study has shown the benefit of TKI based regimen with a mPFS of 11.8 months in trastuzumab-resistant patients (25). For patient recurrent from adjuvant pertuzumab use, pertuzumab resistant was considered regardless of the recurrent time, leading to a change in 1L regimen such as TKI or new anti-HER2 treatment.

For 2L therapy, notable disparities exist between China and the global landscape. The EMILIA study influenced a preference of T-DM1 usage reaching 73% in the US in 2019 (26, 27). In contrast, our data revealed lower ADC usage in China due to the late available of T-DM1, less promising real-world data and a higher incidence rate of grade 3 thrombocytopenia in the Chinese population (28–30). Additionally, some ADC products are still in the clinical trial stage (31). However, because of the PHOEBE study higher efficacy data and earlier approval of pyrotinib (16, 22), a higher prevalence of TKI was observed in the 2L treatment in China.

The real-world efficacy data was slightly lower compared to the randomized clinical trials. Our data showed a 1L median rwPFS of 11.04 months. Dual anti-HER2 mAB regimen for 1L treatment was superior to other regimens, with a median rwPFS of 13.57 months. The number was lower than the data of CLEOPATRA trial (mPFS of 18.5 months) (6), likely due to the lower proportion of patients who had received prior adjuvant treatment, particularly trastuzumab, in the CLEOPATRA study. However, our efficacy results are aligned with the China bridge study, PUFFIN study (mPFS of 14.5 months) (21). A retrospective study from United States reported a real-world PFS of 12.6 or 15 months for pertuzumab-containing regimens in 1L, which is consistent with our finding (32). For 1L TKI + anti-HER2 mAB regimen, the median rwPFS was 12.98 months in our study, lower than the result of the PHILA trial (mPFS of 24.3 months) (17), potentially attributed to the different baselines between our real word study and the phase III study. For example, the PHILA study excluded the patients with a DFI of less than 12 months and those with central nervous system metastases (17). These scenarios were common with pyrotinib treatment in real-world clinical practices. Our data was similar with another real-world study of 1L pyrotinib + trastuzumab + chemotherapy (median rwPFS of 14.46 months) (33), but lower than the result of the PRETTY study (mPFS of 17.8 months) (34). Compared to single TKI or single anti-HER2 mAB, our data showed that combination therapies, such as pertuzumab+ trastuzumab or pyrotinib + trastuzumab, could result in longer rwPFS, and the complementary mechanisms of action led to a more comprehensive HER2 pathway blockade. In our dataset, the small sample size of ADC, potentially applied in cases with higher tumor burden, resulted in unreliable efficacy data. The 1L usage of ADC will be evaluated with anticipation of the results from the DESTINY-Breast 09 trial, which can potentially impact the 1L treatment approach in the future.

This real-world data gave a comprehensive view of the treatment landscape in China with a substantial sample size. However, the retrospective nature brought the potential biases, unbalanced baseline, as well as small sample size in the specific subgroups. The efficiency of follow-up and missing data may also affect result reliability and introduce bias, while the heterogeneity of real-world treatment regimens further complicates comparisons with clinical trial results. Given the dynamic changes in anti-HER2 medications, further research is essential to validate the best treatment choice and sequence as well as predictive and prognostic factors, thereby enhancing precise and personalized treatment strategies.

## 5 Conclusion

This real-world study provided treatment patterns, outcomes, and associated influencing factors, among patients with HER2+ mBC in China, highlighting the notable prevalence of TKI adoption in the country. It elucidated the real-world effectiveness of current anti-HER2 therapy and identified the factors influencing the treatment choice and outcomes, offering valuable guidance for optimizing treatment strategies and achieving personalized patient care in clinical practice.

## Data Availability

The original contributions presented in the study are included in the article/**Supplementary Material**, Further inquiries can be directed to the corresponding author/s.
