# Supplementary material for: Real-world treatment patterns and outcomes among patients with HER2-positive unresectable or metastatic breast cancer in China
Source: Front Oncol. 2025 May 14;15:1527990. doi: 10.3389/fonc.2025.1527990 (PMC12116439; doi:10.3389/fonc.2025.1527990)
Supplement: Supplementary file 1 [file DataSheet1.docx]

Supplemental materials

# Supplementary Table S1 The details of medications by treatment regimens

| Anti-HER2 regimen | No. (%) |
| --- | --- |
| Single anti-HER2 mAB^a^ | N^b^ = 277 |
| Trastuzumab | 231 (83.39) |
| Inetetamab | 44 (15.88) |
| Pertuzumab | 2 (0.72) |
| Dual anti-HER2 mAB | N = 388 |
| Trastuzumab+Pertuzumab | 382 (98.45) |
| Pertuzumab+Inetetamab | 5 (1.29) |
| Trastuzumab+Inetetamab | 1 (0.26) |
| Single TKI | N = 321 |
| Pyrotinib | 306 (95.33) |
| Lapatinib | 12 (3.73) |
| Neratinib | 2 (0.62) |
| Tucatinib | 1 (0.31) |
| TKI+ anti-HER2 mAB | N = 204 |
| Pyrotinib+Trastuzumab | 158 (77.45) |
| Pyrotinib+Inetetamab | 46 (22.55) |
| Pyrotinib+Trastuzumab+Pertuzumab | 6 (2.94) |
| Lapatinib+Trastuzumab | 2 (0.98) |
| Pyrotinib+Lapatinib+Trastuzumab | 1 (0.49) |
| Neratinib+Trastuzumab | 1 (0.49) |
| ADC | N = 114 |
| Trastuzumab Emtansine | 34 (29.82) |
| Disitamab Vedotin | 17 (14.91) |
| ARX788 | 13 (11.40) |
| Pertuzumab+Trastuzumab Deruxtecan | 11 (9.65) |
| Trastuzumab Deruxtecan | 6 (5.26) |
| FS-1502 | 6 (5.26) |
| DX126-262 | 5 (4.39) |
| TAA013 | 5 (4.29) |
| A166 | 4 (3.51) |
| BAT1006 | 3 (2.63) |
| KN026 | 3 (2.63) |
| MRG002 | 2 (1.75) |
| Trastuzumab Emtansine+Pyrotinib | 2 (1.75) |
| Disitamab Vedotin+Pyrotinib | 1 (0.88) |
| DP303c | 1 (0.88) |
| SHR-A1811 | 1 (0.88) |

^a^ Included all treatment lines, with (neo)adjuvant therapies being not contained.

^b^ Number was counted by treatment regimen.

HER2, human epidermal growth factor receptor-2; TKI, tyrosine kinase inhibitor; ADC, antibody-drug conjugate.

# Supplementary Table S2 Treatment regimens in post-line treatment by LOT

| Treatment regimen | No. (%) | 95% CI |
| --- | --- | --- |
| Third-line | N^a^ = 130 |  |
| ADC | 34 (26.15) | (18.84, 34.58) |
| Single TKI | 25 (19.23) | (12.85, 27.07) |
| Single anti-HER2 mAB | 22 (16.92) | (10.92, 24.49) |
| Dual anti-HER2 mAB | 21 (16.15) | (10.29, 23.63) |
| TKI+anti-HER2 mAB | 18 (13.85) | (8.42, 21.00) |
| Non anti-HER2 | 10 (7.69) | (3.75, 13.69) |
| Fourth-line | N = 51 |  |
| Non anti-HER2 | 12 (23.53) | (12.79, 37.49) |
| Single anti-HER2 mAB | 11 (21.57) | (11.29, 35.32) |
| ADC | 10 (19.61) | (9.82, 33.12) |
| Single TKI | 8 (15.69) | (7.02, 28.59) |
| TKI+anti-HER2 mAB | 5 (9.80) | (3.26, 21.41) |
| Dual anti-HER2 mAB | 5 (9.80) | (3.26, 21.41) |
| Fifth-line | N = 18 |  |
| TKI+anti-HER2 mAB | 4 (22.22) | (6.41, 47.64) |
| Single TKI | 4 (22.22) | (6.41, 47.64) |
| ADC | 3 (16.67) | (3.58, 41.42) |
| Single anti-HER2 mAB | 3 (16.67) | (3.58, 41.42) |
| Non anti-HER2 | 3 (16.67) | (3.58, 41.42) |
| Dual anti-HER2 mAB | 1 (5.56) | (0.14, 27.29) |
| Sixth-line | N = 5 |  |
| Single anti-HER2 mAB | 1 (20.00) | (0.51, 71.64) |
| TKI+anti-HER2 mAB | 1 (20.00) | (0.51, 71.64) |
| Non anti-HER2 | 1 (20.00) | (0.51, 71.64) |
| Dual anti-HER2 mAB | 1 (20.00) | (0.51, 71.64) |
| Single TKI | 1 (20.00) | (0.51, 71.64) |
| Seventh-line | N = 1 |  |
| ADC | 1 (100.00) | (2.5, 100.00) |

^a^ The denominator for calculating the composition ratio was the number of patients in each line.

Abbreviations: LOT, line of treatment; CI, confidence interval; ADC, antibody-drug conjugate; TKI, tyrosine kinase inhibitor; HER2, human epidermal growth factor receptor-2.

# Supplementary Table S3 Number of patients who participated in clinical trials by LOT

| Variable | No. (%) | | | | | | | |
| --- | --- | --- | --- | --- | --- | --- | --- | --- |
|  | Total  (N = 1400) | First-line (N = 865) | Second-line (N = 330) | Third-line (N = 130) | Fourth-line (N = 51) | Fifth-line (N = 18) | Sixth-line (N = 5) | Seventh-line (N = 1) |
| Patients participated in clinical trials | 168 (12.00) | 116 (13.41) | 27 (8.18) | 19 (14.62) | 4 (7.84) | 2 (11.11) | 0 (0) | 0 (0) |

Abbreviations: LOT, line of treatment.

# Supplementary Table S4 Univariate analysis for clinical factors of first-line treatment regimen choice

| Variables | No. (%) | | | | | *P* value |
| --- | --- | --- | --- | --- | --- | --- |
|  | TKI (N = 288) | Non-TKI (N = 577) | *P* value | Dual anti-HER2 mAB (N = 312) | Non dual anti-HER2 mAB (N = 553) |  |
| Age group, year |  |  |  |  |  |  |
| <35 | 18 (6.25) | 45 (7.80) | 0.5 | 33 (10.58) | 30 (5.42) | 0.02 |
| [35,65) | 239 (82.99) | 459 (79.55) |  | 241 (77.24) | 457 (82.64) |  |
| ≥65 | 31 (10.76) | 73 (12.65) |  | 38 (12.18) | 66 (11.93) |  |
| Eastern Cooperative Oncology Group performance status | |  |  |  |  |  |
| 0-1 | 187 (97.40) | 308 (96.25) | 0.5 | 154 (95.06) | 341 (97.43) | 0.2 |
| 2-3 | 5 (2.60) | 12 (3.75) |  | 8 (4.94) | 9 (2.57) |  |
| Disease history at initial diagnosis | |  |  |  |  |  |
| De novo mBC | 67 (23.26) | 254 (44.02) | <0.001 | 184 (58.97) | 137 (24.77) | <0.001 |
| Recurrent mBC | 221 (76.74) | 323 (55.98) |  | 128 (41.03) | 416 (75.23) |  |
| Time from the end of (neo)adjuvant treatment to recurrence |  |  |  |  |  |  |
| <6 months | 77 (39.90) | 43 (16.86) | <0.001 | 16 (16.16) | 104 (29.80) | 0.007 |
| ≥6 months | 116 (60.10) | 212 (83.14) |  | 83 (83.84) | 245 (70.20) |  |
| Previous treatment history |  |  |  |  |  |  |
| Anti-HER2 treatment naïve | 135 (46.88) | 438 (75.91) | <0.001 | 245 (78.53) | 328 (59.31) | <0.001 |
| Anti-HER2 pretreated | 153 (53.13) | 139 (24.09) |  | 67 (21.47) | 225 (40.69) |  |
| Previous anti-HER2 treatment in (neo)adjuvant | |  |  |  |  |  |
| Single anti-HER2 mAB | 101 (35.07) | 108 (18.72) | <0.001 | 60 (19.23) | 149 (26.94) | <0.001 |
| Dual anti-HER2 mAB | 50 (17.36) | 23 (3.99) |  | 5 (1.60) | 68 (12.30) |  |
| Other anti-HER2 treatment regimens | 2 (0.69) | 8 (1.39) |  | 2 (0.64) | 8 (1.45) |  |
| Visceral Disease |  |  |  |  |  |  |
| Yes | 169 (58.68) | 379 (66.14) | 0.04 | 199 (64.61) | 349 (63.11) | 0.03 |
| No | 119 (41.32) | 194 (33.86) |  | 109 (35.39) | 204 (36.89) |  |
| Brain metastasis |  |  |  |  |  |  |
| Yes | 55 (19.10) | 40 (6.93) | <0.001 | 15 (4.81) | 80 (14.47) | <0.001 |
| No | 233 (80.90) | 537 (93.07) |  | 297 (95.19) | 473 (85.53) |  |
| Number of brain metastatic lesion | |  |  |  |  |  |
| Single | 15 (5.21) | 12 (2.08) | <0.001 | 5 (1.60) | 22 (3.98) | <0.001 |
| Multiple | 29 (10.07) | 24 (4.16) |  | 8 (2.56) | 45 (8.14) |  |
| Unknown | 11 (3.82) | 4 (0.69) |  | 2 (0.64) | 13 (2.35) |  |
| Non-brain metastasis | 233 (80.90) | 537 (93.07) |  | 297 (95.19) | 473 (85.53) |  |

Abbreviations: TKI, tyrosine kinase inhibitor; HER2, human epidermal growth factor receptor 2; mBC, metastatic breast cancer.

# Supplementary Table S5 Disease progression of different treatment lines among the total patients (from second-line to seventh-line)

| Treatment regimen | Number of patients | Disease progression or death, No. (%) | Censor,  No. (%) | rwPFS^a^ (95% CI), month |
| --- | --- | --- | --- | --- |
| Second-line | 265 | 142 (53.58) | 123 (46.42) | 7.59 (6.21, 9.20) |
| Single TKI | 93 | 50 (53.76) | 43 (46.24) | 7.85 (6.21, 11.96) |
| Single anti-HER2 mAB | 44 | 24 (54.55) | 20 (45.45) | 5.42 (3.88, NA) |
| Dual anti-HER2 mAB | 42 | 21 (50.00) | 21 (50.00) | 9.23 (7.39, NA) |
| TKI+anti-HER2 mAB | 39 | 18 (46.15) | 21 (53.85) | 9.60 (4.07, NA) |
| ADC | 30 | 17 (56.67) | 13 (43.33) | 4.70 (3.71, NA) |
| Non anti-HER2 | 17 | 12 (70.59) | 5 (29.41) | 5.00 (2.43, NA) |
| Third-line | 106 | 54 (50.94) | 52 (49.06) | 4.53 (4.27, 6.61) |
| ADC | 28 | 15 (53.57) | 13 (46.43) | 4.53 (2.04, NA) |
| Dual anti-HER2 mAB | 20 | 10 (50.00) | 10 (50.00) | 9.27 (4.34, NA) |
| Single TKI | 19 | 12 (63.16) | 7 (36.84) | 4.21 (3.52, NA) |
| Single anti-HER2 mAB | 15 | 6 (40.00) | 9 (60.00) | 6.08 (2.76, NA) |
| TKI+anti-HER2 mAB | 14 | 4 (28.57) | 10 (71.43) | NA (4.27, NA) |
| Non anti-HER2 | 10 | 7 (70.00) | 3 (30.00) | 3.04 (0.95, NA) |
| Fourth-line | 35 | 20 (57.14) | 15 (42.86) | 3.98 (3.22, 5.36) |
| Non anti-HER2 | 8 | 6 (75.00) | 2 (25.00) | 2.46 (1.58, NA) |
| Single anti-HER2 mAB | 7 | 6 (85.71) | 1 (14.29) | 4.04 (1.81, NA) |
| Single TKI | 7 | 1 (14.29) | 6 (85.71) | NA (NA, NA) |
| ADC | 5 | 2 (40.00) | 3 (60.00) | 3.98 (3.32, NA) |
| Dual anti-HER2 mAB | 4 | 2 (50.00) | 2 (50.00) | 2.04 (1.05, NA) |
| TKI+anti-HER2 mAB | 4 | 3 (75.00) | 1 (25.00) | 3.52 (2.73, NA) |
| Fifth-line | 12 | 5 (41.67) | 7 (58.33) | 3.71 (2.69, NA) |
| TKI+anti-HER2 mAB | 4 | 2 (50.00) | 2 (50.00) | 4.47 (1.35, NA) |
| ADC | 2 | 0 (0) | 2 (100) | NA (NA, NA) |
| Non anti-HER2 | 2 | 1 (50.00) | 1 (50.00) | 3.71 (NA, NA) |
| Single TKI | 2 | 0 (0) | 2 (100.00) | NA (NA, NA) |
| Dual anti-HER2 mAB | 1 | 1 (100.00) | 0 (0) | 2.69 (NA, NA) |
| Single anti-HER2 mAB | 1 | 1 (100.00) | 0 (0) | 2.92 (NA, NA) |
| Sixth-line | 3 | 1 (33.33) | 2 (66.67) | NA (1.81, NA) |
| Dual anti-HER2 mAB | 1 | 0 (0) | 1 (100.00) | NA (NA, NA) |
| Single anti-HER2 mAB | 1 | 1 (100.00) | 0 (0) | 1.81 (NA, NA) |
| Single TKI | 1 | 0 (0) | 1 (100.00) | NA (NA, NA) |
| ADC | 1 | 0 (0) | 1 (100.00) | NA (NA, NA) |
| Seventh-line | 1 | 0 (0) | 1 (100.00) | NA (NA, NA) |

^a^ The number of patients only included those who had PFS related records; Among them, the number of cases with data missing from second-line treatment to seventh-line treatment was 65, 24, 16, 6, 2, and 0 in sequence.

Abbreviations: rwPFS, real-world progression-free survival; CI, confidence interval; NA, not available; TKI, tyrosine kinase inhibitor; HER2, human epidermal growth factor receptor-2; ADC, antibody-drug conjugate.

# Supplementary Table S6 Disease progression of second-line treatment following different first-line treatment regimens

| First-line treatment | Second-line treatment | Number of patients | Disease progression or death,  No. (%) | Censor,  No. (%) | rwPFS for second-line treatment (95% CI), month | rwPFS from first-line to second-line treatment (95% CI), month |
| --- | --- | --- | --- | --- | --- | --- |
| Single anti-HER2 mAB | Single TKI | 38 | 21 (55.26) | 17 (44.74) | 9.00 (7.16,NA) | 20.97 (15.28,NA) |
| Single anti-HER2 mAB | TKI+anti-HER2 mAB | 19 | 8 (42.11) | 11 (57.89) | 6.31 (3.68,NA) | 18.44 (11.53,NA) |
| Single anti-HER2 mAB | Single anti-HER2 mAB | 12 | 7 (58.33) | 5 (41.67) | 7.43 (4.53,NA) | 16.17 (12.95,NA) |
| Single anti-HER2 mAB | Dual anti-HER2 mAB | 7 | 4 (57.14) | 3 (42.86) | 9.92 (9.23,NA) | 13.60 (11.63,NA) |
| Single anti-HER2 mAB | Non anti-HER2 | 5 | 4 (80.00) | 1 (20.00) | 8.35 (2.89,NA) | 17.12 (14.03,NA) |
| Single anti-HER2 mAB | ADC | 4 | 2 (50.00) | 2 (50.00) | 11.67 (7.66,NA) | 23.66 (12.06,NA) |
| Dual anti-HER2 mAB | Single TKI | 36 | 16 (44.44) | 20 (55.56) | 7.62 (5.55,NA) | 24.32 (22.05,NA) |
| Dual anti-HER2 mAB | TKI+anti-HER2 mAB | 13 | 5 (38.46) | 8 (61.54) | 9.60 (4.93,NA) | NA (11.47,NA) |
| Dual anti-HER2 mAB | Dual anti-HER2 mAB | 8 | 4 (50.00) | 4 (50.00) | 7.39 (3.75,NA) | 14.53 (8.54,NA) |
| Dual anti-HER2 mAB | ADC | 7 | 3 (42.86) | 4 (57.14) | 13.80 (4.60,NA) | 22.97 (9.69,NA) |
| Dual anti-HER2 mAB | Non anti-HER2 | 4 | 3 (75.00) | 1 (25.00) | 2.23 (1.51,NA) | 9.43 (7.07,NA) |
| Dual anti-HER2 mAB | Single anti-HER2 mAB | 3 | 2 (66.67) | 1 (33.33) | 9.99 (0.79,NA) | 20.54 (5.72,NA) |
| Single TKI | Single anti-HER2 mAB | 17 | 10 (58.82) | 7 (41.18) | 3.02 (2.76,NA) | 13.87 (11.99,NA) |
| Single TKI | ADC | 13 | 9 (69.23) | 4 (30.77) | 4.40 (2.60,NA) | 16.14 (9.33,NA) |
| Single TKI | Dual anti-HER2 mAB | 13 | 8 (61.54) | 5 (38.46) | 7.62 (3.09,NA) | 18.99 (14.10,NA) |
| Single TKI | Non anti-HER2 | 4 | 2 (50.00) | 2 (50.00) | 6.05 (0.99,NA) | 14.59 (9.66,NA) |
| Single TKI | TKI+anti-HER2 mAB | 3 | 2 (66.67) | 1 (33.33) | 3.65 (2.33,NA) | 22.97 (8.94,NA) |
| Single TKI | Single TKI | 2 | 1 (50.00) | 1 (50.00) | 7.23 (NA,NA) | 9.00 (9.00,NA) |
| TKI+anti-HER2 mAB | Single TKI | 7 | 4 (57.14) | 3 (42.86) | 3.78 (1.48,NA) | 16.10 (11.73,NA) |
| TKI+anti-HER2 mAB | ADC | 5 | 3 (60.00) | 2 (40.00) | 2.46 (1.84,NA) | 14.10 (10.15,NA) |
| TKI+anti-HER2 mAB | Dual anti-HER2 mAB | 5 | 3 (60.00) | 2 (40.00) | 7.59 (2.40,NA) | 21.31 (8.97,NA) |
| TKI+anti-HER2 mAB | Single anti-HER2 mAB | 5 | 2 (40.00) | 3 (60.00) | NA (4.67,NA) | 21.85 (21.85,NA) |
| TKI+anti-HER2 mAB | TKI+anti-HER2 mAB | 4 | 3 (75.00) | 1 (25.00) | 9.63 (1.22,NA) | 13.13 (9.50,NA) |
| TKI+anti-HER2 mAB | Non anti-HER2 | 1 | 0 (0) | 1 (100.00) | NA (NA,NA) | NA (NA,NA) |
| Non anti-HER2 | Dual anti-HER2 mAB | 8 | 2 (25.00) | 6 (75.00) | 8.97 (8.97,NA) | NA (13.28,NA) |
| Non anti-HER2 | Single TKI | 7 | 6 (85.71) | 1 (14.29) | 6.05 (0.89,NA) | 12.22 (4.57,NA) |
| Non anti-HER2 | Single anti-HER2 mAB | 6 | 2 (33.33) | 4 (66.67) | NA (3.88,NA) | NA (6.41,NA) |
| Non anti-HER2 | Non anti-HER2 | 3 | 3 (100.00) | 0 (0) | 5.00 (0.72,NA) | 8.54 (6.67,NA) |
| ADC | Single TKI | 3 | 2 (66.67) | 1 (33.33) | 3.34 (2.23,NA) | 7.38 (6.57,NA) |
| ADC | ADC | 1 | 0 (0) | 1 (100.00) | NA (NA,NA) | NA (NA,NA) |
| ADC | Dual anti-HER2 mAB | 1 | 0 (0) | 1 (100.00) | NA (NA,NA) | NA (NA,NA) |
| ADC | Single anti-HER2 mAB | 1 | 1 (100.00) | 0 (0) | 1.38 (NA,NA) | 8.38 (NA,NA) |

Abbreviations: rwPFS, real-world progression-free survival; CI, confidence interval; NA, not available; HER2, human epidermal growth factor receptor-2; TKI, tyrosine kinase inhibitor; ADC, antibody-drug conjugate.

# Supplementary Table S7 Univariate analysis for clinical factors and the progression free survival of first-line treatment

| Variables | Number of patients | Progression or death | HR (95%CI) | *P* value |
| --- | --- | --- | --- | --- |
| TKI-based regimen |  |  |  |  |
| Non-TKI | 484 | 243 | 1 | 0.60 |
| TKI | 242 | 142 | 1.05 (0.85, 1.29) |  |
| Dual anti-HER2 mAB-based regimen |  |  |  |  |
| Dual anti-HER2 mAB | 255 | 98 | 1 | <0.001 |
| Non dual anti-HER2 mAB | 471 | 287 | 1.72 (1.36, 2.16) |  |
| Age group, year |  |  |  |  |
| <35 | 53 | 24 | 1 | NA |
| [35,65) | 592 | 319 | 1.30 (0.86, 1.97) | 0.20 |
| ≥65 | 81 | 42 | 1.36 (0.82, 2.25) | 0.20 |
| Eastern Cooperative Oncology Group performance status |  |  |  |  |
| 0-1 | 423 | 245 | 1 | 0.90 |
| 2-3 | 16 | 9 | 1.06 (0.54, 2.06) |  |
| Disease history at initial diagnosis |  |  |  |  |
| De novo mBC | 270 | 130 | 1 | 0.05 |
| Recurrent mBC | 456 | 255 | 1.24 (1.00, 1.53) |  |
| Time from the end of (neo)adjuvant treatment to recurrence |  |  |  |  |
| <6 months | 103 | 68 | 1 | 0.01 |
| ≥6 months | 274 | 156 | 0.69 (0.52, 0.91) |  |
| Previous treatment history |  |  |  |  |
| Anti-HER2 treatment naïve | 485 | 244 | 1 | 0.05 |
| Anti-HER2 pretreated | 241 | 141 | 1.23 (1.00, 1.51) |  |
| Previous anti-HER2 treatment in (neo)adjuvant |  |  |  |  |
| Single anti-HER2 mAB | 175 | 100 | 1 | NA |
| Dual anti-HER2 mAB | 56 | 37 | 1.93 (1.32, 2.83) | <0.001 |
| Other anti-HER2 treatment regimens | 10 | 4 | 1.33 (0.49, 3.61) | 0.60 |
| Visceral disease |  |  |  |  |
| Yes | 461 | 255 | 1.16 (0.94, 1.43) | 0.20 |
| No | 262 | 127 | 1 |  |
| Brain metastasis |  |  |  |  |
| Yes | 83 | 41 | 0.97 (0.70, 1.34) | 0.80 |
| No | 643 | 344 | 1 |  |
| Number of brain metastatic lesions |  |  |  |  |
| Single | 26 | 15 | 1 | NA |
| Multiple | 43 | 18 | 0.60 (0.30, 1.18) | 0.14 |
| Unknown | 14 | 8 | 1.26 (0.53, 2.97) | 0.60 |
| Non brain metastasis | 643 | 344 | 0.82 (0.49, 1.38) | 0.50 |

Abbreviations: HR, hazard ratio; CI, confidence interval; NA, not applicable; TKI, tyrosine kinase inhibitor; mBC, metastatic breast cancer; HER2, human epidermal growth factor receptor 2.


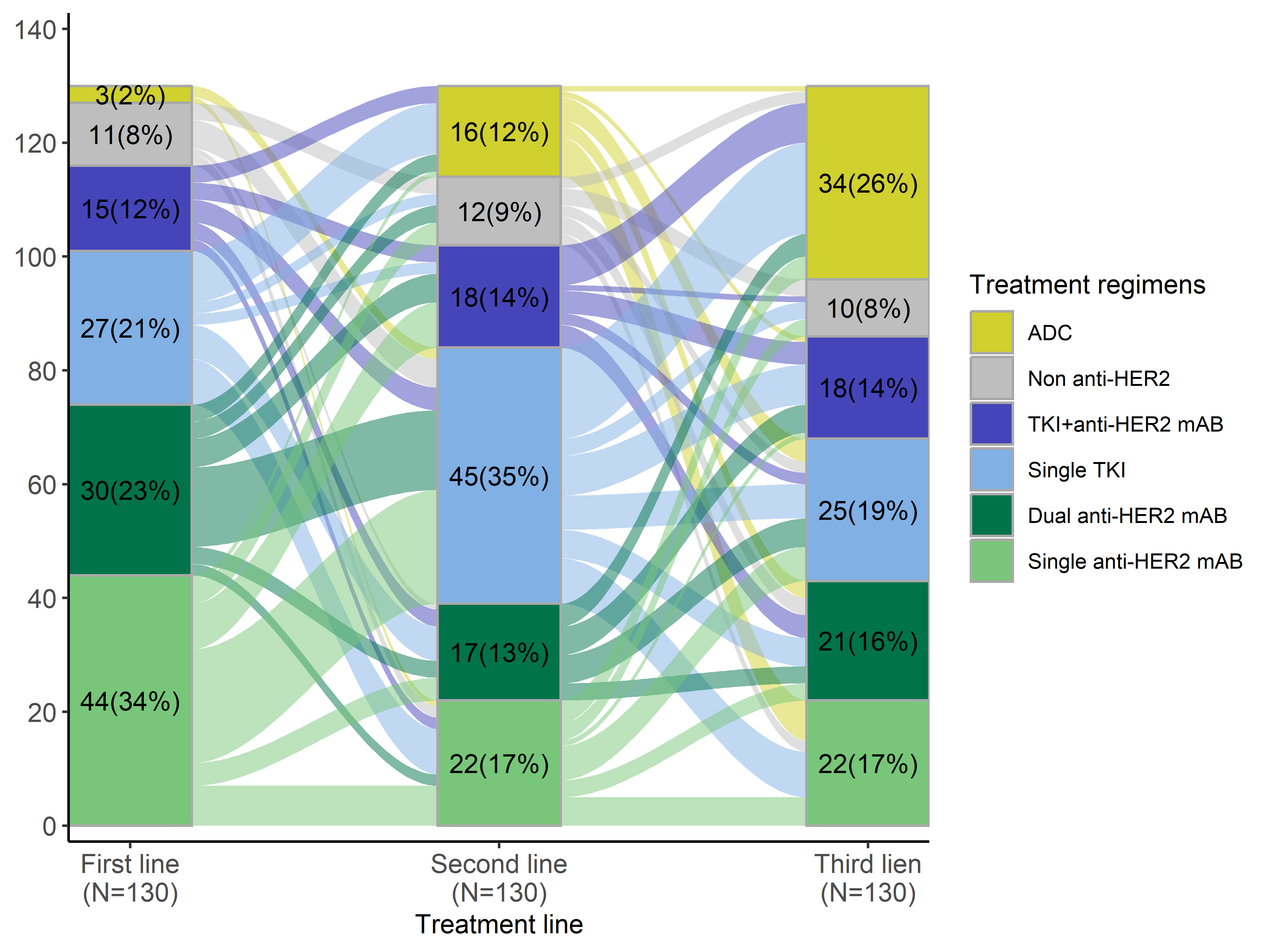


|  |  | Second-line No. (%) | | | | | | Total  No. (%) |
| --- | --- | --- | --- | --- | --- | --- | --- | --- |
|  |  | Single anti-HER2 mAB | Dual anti-HER2 mAB | TKI+anti-HER2 mAB | Single TKI | Non anti-HER2 | ADC |  |
| First-line No. (%) | Single anti-HER2 mAB | 7 (5.38) | 4 (3.08) | 8 (6.15) | 20 (15.38) | 4 (3.08) | 1 (0.77) | 44 (33.85) |
|  | Dual anti-HER2 mAB | 2 (1.54) | 3 (2.31) | 5 (3.85) | 14 (10.77) | 3 (2.31) | 3 (2.31) | 30 (23.08) |
|  | TKI+anti-HER2 mAB | 2 (1.54) | 3 (2.31) | 3 (2.31) | 4 (3.08) | 0 (0) | 3 (2.31) | 15 (11.54) |
|  | Single TKI | 8 (6.15) | 6 (4.62) | 2 (1.54) | 0 (0) | 2 (1.54) | 9 (6.92) | 27 (20.77) |
|  | Non anti-HER2 | 2 (1.54) | 1 (0.77) | 0 (0) | 5 (3.85) | 3 (2.31) | 0 (0) | 11 (8.46) |
|  | ADC | 1 (0.77) | 0 (0) | 0 (0) | 2 (1.54) | 0 (0) | 0 (0) | 3 (2.31) |
| Total  No. (%) | | 22 (16.92) | 17 (13.08) | 18 (13.85) | 45 (34.62) | 12 (9.23) | 16 (12.31) | 130 (100.00) |
|  |  | Third-line No. (%) | | | | | | Total  No. (%) |
|  |  | Single anti-HER2 mAB | Dual anti-HER2 mAB | TKI+anti-HER2 mAB | Single TKI | Non-anti-HER2 | ADC |  |
| Second-line No. (%) | Single anti-HER2 mAB | 5 (3.85) | 3 (2.31) | 1 (0.77) | 6 (4.62) | 3 (2.31) | 4 (3.08) | 22 (16.92) |
|  | Dual anti-HER2 mAB | 0 (0) | 3 (2.31) | 5 (3.85) | 5 (3.85) | 0 (0) | 4 (3.08) | 17 (13.08) |
|  | TKI+anti-HER2 mAB | 0 (0) | 4 (3.08) | 4 (3.08) | 2 (1.54) | 1 (0.77) | 7 (5.38) | 18 (13.85) |
|  | Single TKI | 8 (6.15) | 5 (3.85) | 7 (5.38) | 6 (4.62) | 3 (2.31) | 16 (12.31) | 45 (34.62) |
|  | Non anti-HER2 | 2 (1.54) | 3 (2.31) | 0 (0) | 2 (1.54) | 3 (2.31) | 2 (1.54) | 12 (9.23) |
|  | ADC | 7 (5.38) | 3 (2.31) | 1 (0.77) | 4 (3.08) | 0 (0) | 1 (0.77) | 16 (12.31) |
| Total  No. (%) | | 22 (16.92) | 21 (16.15) | 18 (13.85) | 25 (19.23) | 10 (7.69) | 34 (26.15) | 130 (100.00) |

# Figure S1 Treatment sequence of patients with advanced breast cancer who received at least three lines of systemic treatment.

# HER2, human epidermal growth factor receptor-2; TKI, tyrosine kinase inhibitor; ADC, antibody-drug conjugate.


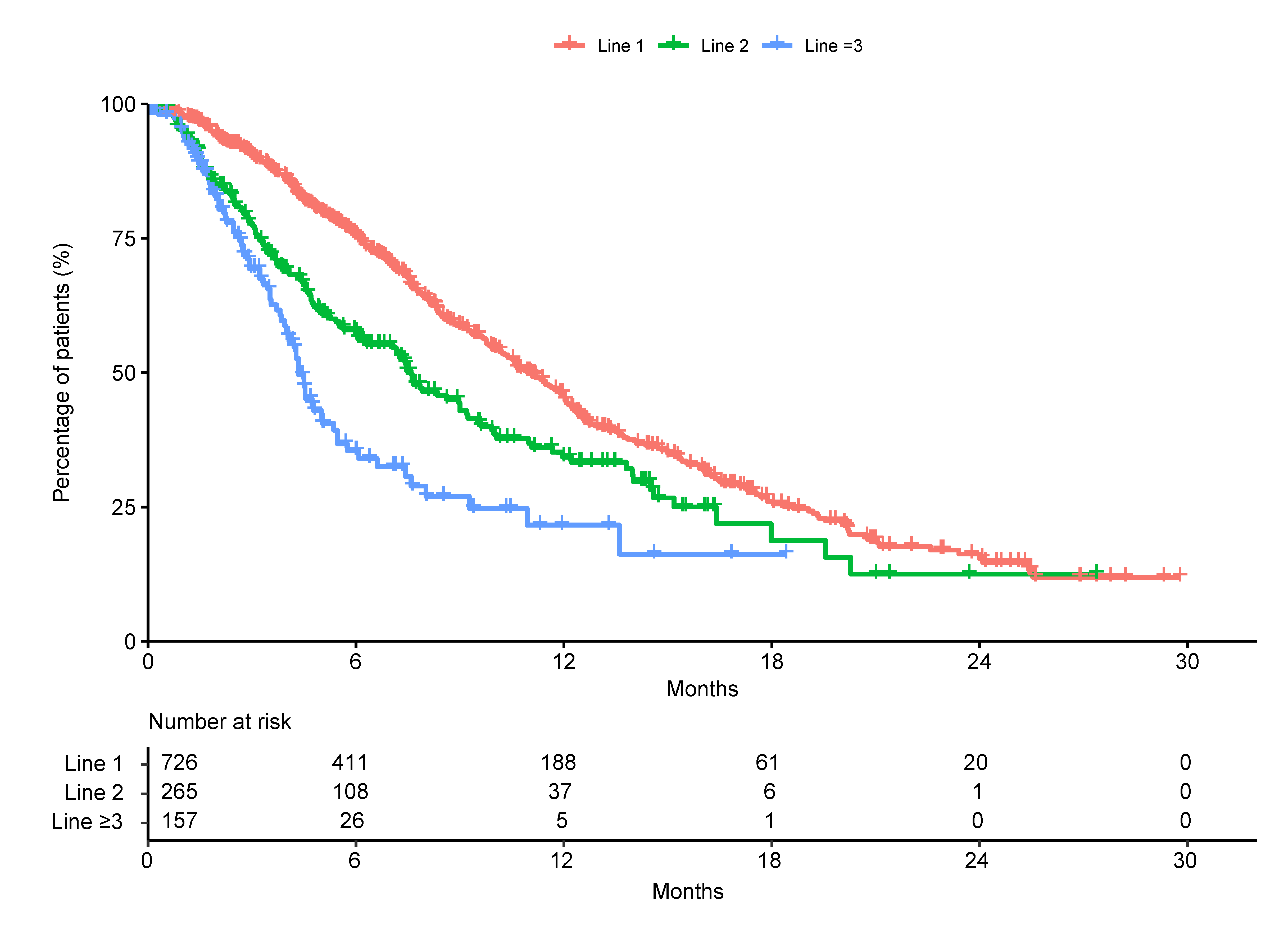


# Figure S2 Progression-free survival of treatment according to treatment line.
